# Sustained-release methylphenidate in methamphetamine dependence treatment: a double-blind and placebo-controlled trial

**DOI:** 10.1186/s40199-015-0092-y

**Published:** 2015-01-15

**Authors:** Farzin Rezaei, Maryam Emami, Shakiba Zahed, Mohammad-Javad Morabbi, Mohammadhadi Farahzadi, Shahin Akhondzadeh

**Affiliations:** Department of psychiatry, Kurdistan University of Medical Sciences, Sanandaj, Iran; Department of Health Education and Health Promotion, Faculty of Health, Isfahan University of Medical Sciences, Isfahan, Iran; Department of Neuroscience, School of Advanced Medical Technologies, Tehran University of Medical Sciences, Tehran, Iran; Psychiatric Research Center, Roozbeh Hospital, Tehran University of Medical Sciences, South Kargar Street, Tehran, 13337 Iran

**Keywords:** Clinical trial, Dependence, Methamphetamine, Methylphenidate

## Abstract

**Background:**

The objective of this randomized, double-blind, placebo-controlled study was to evaluate the efficacy of sustained-release methylphenidate (MPH-SR) in treatment of methamphetamine dependence.

**Methods:**

Fifty-six individuals who met DSM-IV-TR criteria for methamphetamine dependence participated in this 10-week trial. The participants were randomly allocated into two groups and received 18 to 54 mg/day sustained-released methylphenidate or placebo for 10 weeks. Craving was evaluated by a visual analogue craving scale every week. Urinary screening test for methamphetamine was carried out each week. The Beck Depression Inventory-II (BDI-II) was used to monitor participant depressive symptoms at baseline and bi-weekly during the treatment period.

**Results:**

At the end of the trial, the MPH-SR group was less methamphetamine positive compared to the placebo group and the difference was significant (p = 0.03). By the end of the study, MPH-SR group showed significantly less craving scores compared to the placebo group [MD (95% CI) = -10.28(0.88-19.18), t(54) = 2.19, p = 0.03]. There was greater improvement in the depressive symptoms scores in the intervention group compared to the placebo group [MD (95% CI) =2.03(0.31-3.75), t (54) =2.37, p = 0.02].

**Conclusion:**

Sustained-released methylphenidate was safe and well tolerated among active methamphetamine users and significantly reduced methamphetamine use, craving and depressive symptoms.

**Trial registration:**

IRCT201202281556N38

## Background

Methamphetamine is a psychostimulant that is highly addictive and affects monoamine neurotransmitter systems [1]. Methamphetamine and related stimulants are the second most frequently used illicit drugs worldwide, second only to cannabis. It is estimated that more than 35 million people around the world use this class of substance [2–4]. Methamphetamine dependence is associated with a number of psychiatric disorders including depression and psychosis [5–7]. Furthermore, methamphetamine use is accompanied with various medical consequences such as myocardial infarction, renal failure, cerebral hemorrhage, muscle damage, nasal and sinus damage and sudden death [8–12]. Methamphetamine abuse and dependence have become a major health problem imposing a great burden on the society [13–15]. In recent years, a dramatic rise in methamphetamine use has occurred in many countries [16]. There is evidence for significantly increased prevalence of methamphetamine abuse in Iran in past years [17].

In the last decade, many medications have been used for treatment of methamphetamine dependence including modafinil, antidepressants, ondansetron, risperidone, aripiprazole, baclofen, topiramate, N-acetyl cysteine, naltrexone, and gabapentin, but none demonstrated consistent efficacy [2,10,13,18–25]. Some studies suggested sustained-release dextroamphetamine and methylphenidate as effective pharmacotherapy for methamphetamine dependence [26–30]. Given that methylphenidate antagonizes the effects of methamphetamine *in vitro*, some researchers have tried it as a potential candidate for treatment of methamphetamine dependence [31,32]. The results of some preliminary studies have demonstrated that a maintenance pharmacotherapy program of daily sustained-release amphetamine could increase retention of patients and decrease relapse rate of methamphetamine dependence [33] although a study has findings to the contrary [34]. Some studies questioned the notion of replacement therapy for amphetamine dependence (a cochrane review) [35]. The objective of this randomized, double-blind, placebo-controlled study was to evaluate the efficacy of sustained-released methylphenidate in treatment of methamphetamine dependence.

## Methods

### Trial design and setting

This study was a double-blind, randomized, placebo-controlled trial. The participants (consecutive patients screened for the trial) were men and women with methamphetamine dependence attending outpatient clinics in Sanandaj and Tehran from June 2013 to August 2014.

### Participants

Inclusion criteria were diagnosis of methamphetamine dependence based on DSM-IV-TR between 18 to 65 years of age, and positive methamphetamine urine test at the beginning of the study. Participants met none of the following exclusion criteria: (1) any other mental disorder on axis I except for depression, (2) any serious medical or neurological problem (3), IQ <70, (4) abuse of other substances except for nicotine and methadone over the last six months; (5) history of Attention Defecit-Hyperactivity Disorder (ADHD) during childhood, (6) pregnancy and breast feeding, (7) development of psychotic symptoms requiring pharmacotherapy and (8) serious suicidal ideations. The study protocol was approved by the Institutional Review Board of Tehran University of Medical Sciences (Grant No: 16507) and was performed in accordance with the Declaration of Helsinki and its subsequent revisions. Patients gave written informed consent before entry into the study. Patients were informed they are free to withdraw from the study at any time, without giving a reason. The trial was registered at the Iranian registry of clinical trials (www.irct.ir; registration number: IRCT201202281556N38) prior to conducting the study.

### Interventions

Fifty-six individuals who met DSM-IV-TR criteria for methamphetamine dependence participated in this 10-week trial. The participants were randomly allocated into two groups. Group 1 received 18 mg/day sustained-released methylphenidate during the first week and 36 mg/day during the second week and then received 54 mg/day sustained-released methylphenidate for the remaining 8 weeks. Group 2 received placebo for 10 weeks. The medication was given daily under staff supervision.

### Outcome

Our primary outcome was Methamphetamine craving. Craving was evaluated by a visual analogue craving scale every week that ranges from 0 (no cravings) to 100 (most intense cravings possible) [22]. Data was gathered by a demographic questionnaire and severity of addiction was assessed by Addiction Severity Index (ASI) at the beginning of the study. Several studies have shown that ASI had acceptable reliability and validity [36–38]. It has been reported that the Persian version of this inventory has good reliability and validity [38,39]. Urinary screening test for methamphetamine was carried out each week Urine samples were analyzed using radioimmunoassay (Phamatech, Inc, San Diego, CA) for qualitative tests of MA metabolite. The Beck Depression Inventory II (BDI-II) rating scale was used to monitor participant depressive symptoms at baseline and bi-weekly during the treatment period [40]. Medication adherence was measured using weekly tablet counts justified against participant reports of medication taking to calculate the proportion of dispensed medication doses that were taken.

### Sample size

Assuming a clinically significant difference of 4 on the visual analogue craving scale score between MPH-SR and placebo groups with a standard deviation (SD) of 4.5 (based on our pilot study), a power of 80%, and a two-sided significance level of 5%, a minimal sample size of 40 was estimated. Considering an attrition rate of 10%, a total sample size of 48 was planned.

### Randomization, allocation, concealment and blinding

Generation of randomization codes was conducted by permuted randomization block using Excel software (blocks of four, allocation ratio 1:1). Randomization was performed by an independent party who was not involved elsewhere in the trial. Concealment of allocation was performed using sequentially numbered, sealed, opaque, and stapled envelopes. Separate persons were responsible for generation of randomization codes, treatment allocation and interviewing. The patients, research investigators, nurses and interviewers were all blinded to the treatment allocation. MPH-SR and placebo were completely identical in their size, color, shape, texture and odor.

### Statistical analysis

Mean differences between the groups were reported as mean (95% confidence intervals (95% CI). All analyses were based on the intention-to-treat sample and were performed using the last observation carried forward (LOCF) procedure. To compare the score and the behavior of the two treatment groups over the course of the trial, two-factor repeated measure analysis of variance (ANOVA) was used. Greenhouse Geisser’s correction was used whenever Mauchly’s test of sphericity was significant. Comparison of score change from baseline to each time point between the two groups was done using the unpaired t-test. A p value of <0.05 was considered significant.

## Results

### Basic characteristics

**Figure 1:**
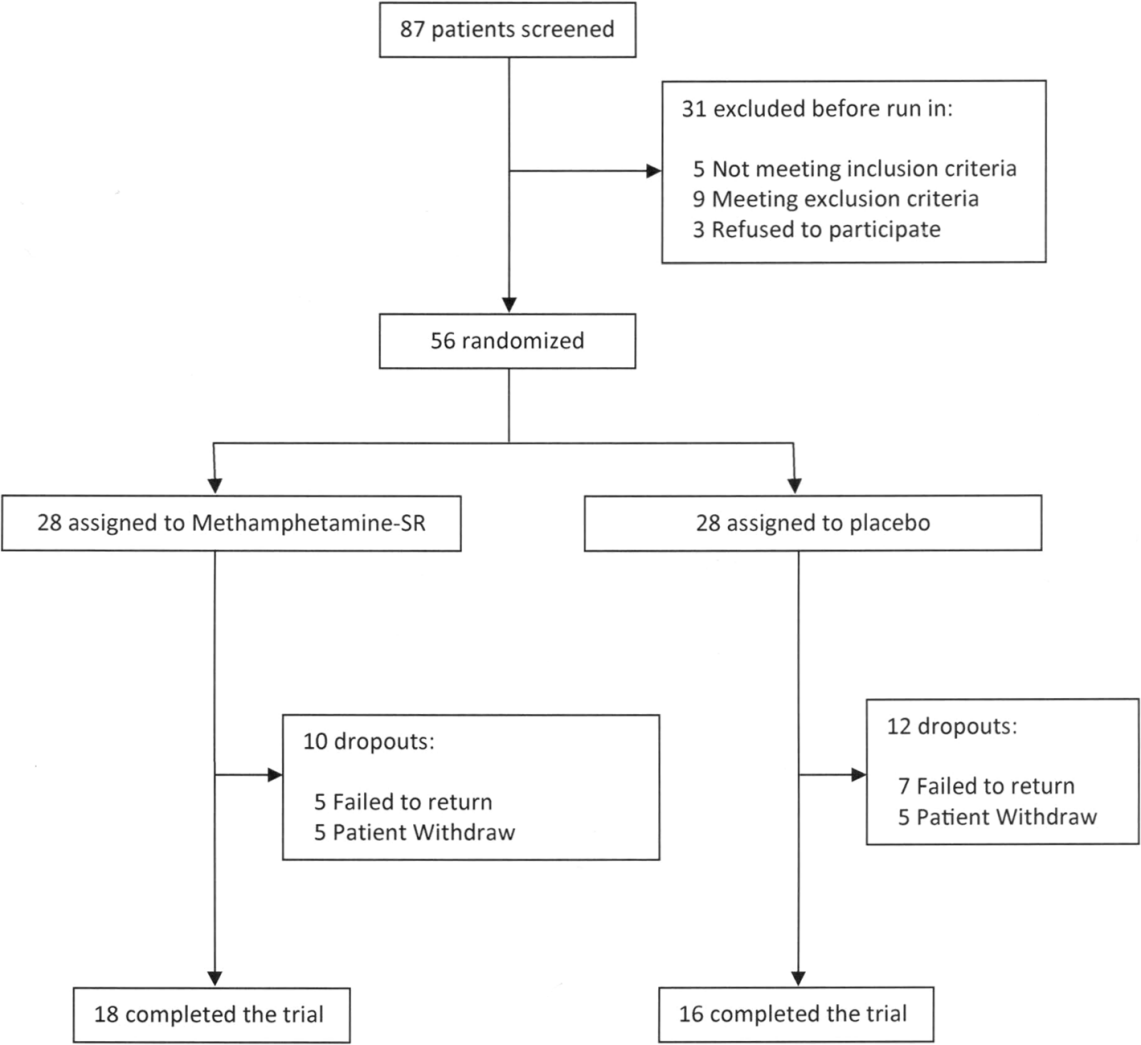
**Flow diagram of the study.**

Eighty-seven patients were screened to participate in this study and a total number of 56 patients were entered into the study. Ten patients from the MPH-SR group and 12 patients from the placebo group dropped out before week 6 and 34 patients completed the trial (Figure 1). Baseline psychiatric characteristics, demographic and drug use of both groups were compared yielding no significance between the two groups (Table 1).

**Table 1:** **Baseline characteristics of the participants, drug use and psychiatric characteristics**

	**Mean (SD) or N**	**Mean (SD) or N**	**P value**
	**Methylphenidate slow release (N = 28)**	**Placebo (N = 28)**	
**Age (years)**	35.6(10.8)	34.7(11.5)	ns
**Gender, n**	8	7	ns
• **Female**	20	21	
• **Male**			
**Marital status, n**			ns
• **Single**	18	19	
• **Married**	6	5	
• **Divorced**	4	4	
**Level of education, n**			ns
• **Illiterate**	2	1	
• **Primary school**	16	17	
• **High school diploma**	8	9	
• **University degree**	2	1	
**Smoking, n**	25	26	ns
**Use of methadone**	12	11	ns
**Employed, n**	5	7	ns
**Years of methamphetamine use**	13.3(8.5)	12.8(9.1)	ns
**Days of methamphetamine use (past month)**	10.2(8.7)	10.4(8.8)	ns
**Route of methamphetamine use, n**			ns
• **Smoking**	23	22	
• **Nasal**	3	2	
• **Injection**	1	1	
• **Oral**	1	3	
**Addiction severity index composite score**			ns
• **Medical**	0.25(0.31)	0.28(0.26)	
• **Employment**	0.22(017)	0.24(0.19)	
• **Alcohol**	0.15(0.10)	0.14(0.13)	
• **Drug**	0.28(0.1)	0.29(0.12)	
• **Legal**	0.08(0.17)	0.07(0.16)	
• **Family/Social**	0.22(0.23)	0.19(0.20)	
• **Psychiatric**	0.24(0.25)	0.22(0.23	

N: number; SD: Standard Deviation; ns: non-significant.

### Urine drug screen results

**Figure 2:**
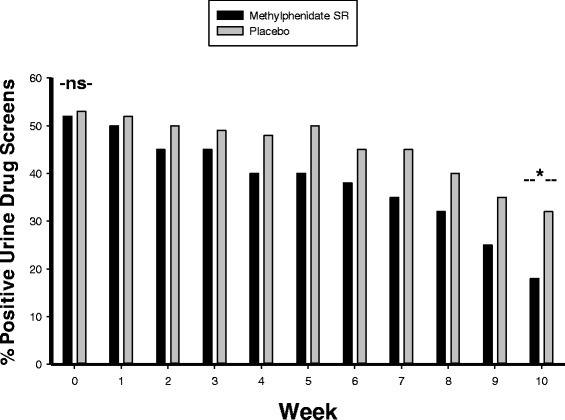
**Positive urine drug screens in the two groups (%).** NS indicates non-significant and * = p < 0.05.

There was no significant difference between the two groups in baseline methamephetamine positive urine drug screen tests. Nevertheless, at week 10, the MPH-SR group was less methamphetamine positive compare to the placebo and the difference was significant (P = 0.03) (Figure 2).

### Methamphetamine craving

**Figure 3:**
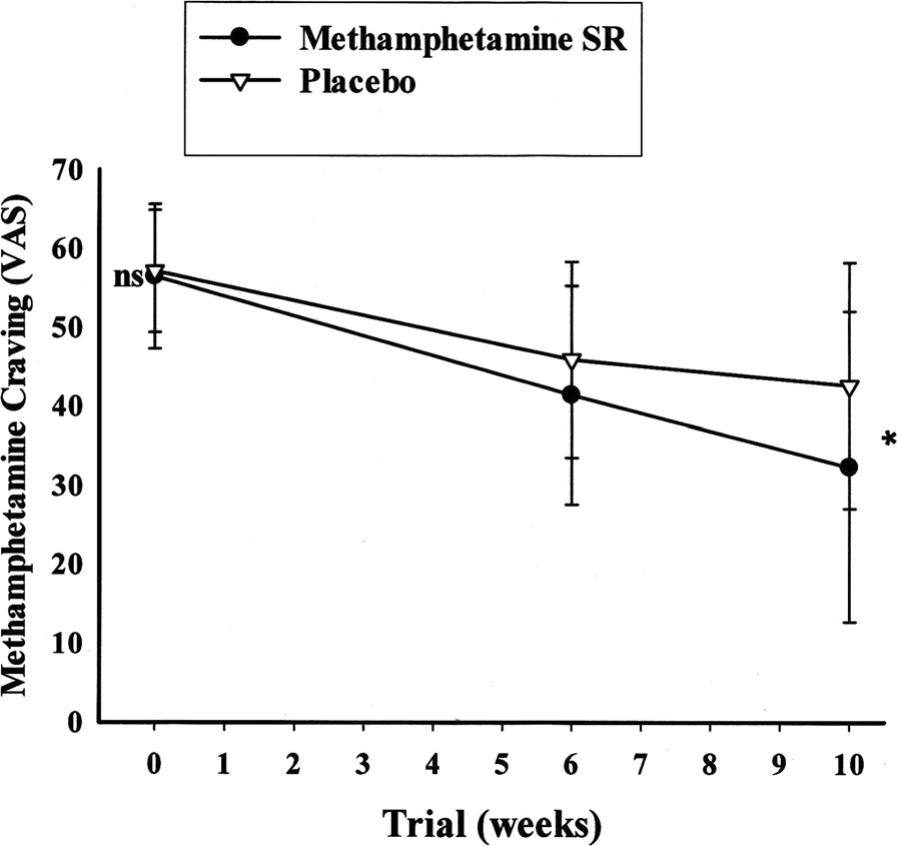
**Repeated measure for comparison of the effects of two treatments on methamphetamine craving score.** Values represent mean ± standard deviation of the mean. P-values demonstrate the result of the independent T-test for comparison of scores between two treatment groups at each time point. NS indicates non-significant; and * = p < 0.05.

Baseline Methamphetamine craving scores were not significantly different between the treatment groups [MD (95% CI) = 0.57(-3.97 to 5.12), t(54) = 0.25, p = 0.39]. At the study endpoint, the result of repeated measure analysis demonstrated a significant effect of time × treatment interaction [F = 4.06, p = 0.046] (Figure 3). By the end of the trial, MPH-SR group showed significantly less craving scores compared to the placebo group [MD (95% CI) = -10.28(0.88-19.18), t(54) = 2.19, p = 0.03] (Figure 3).

### Depressive symptoms

**Figure 4:**
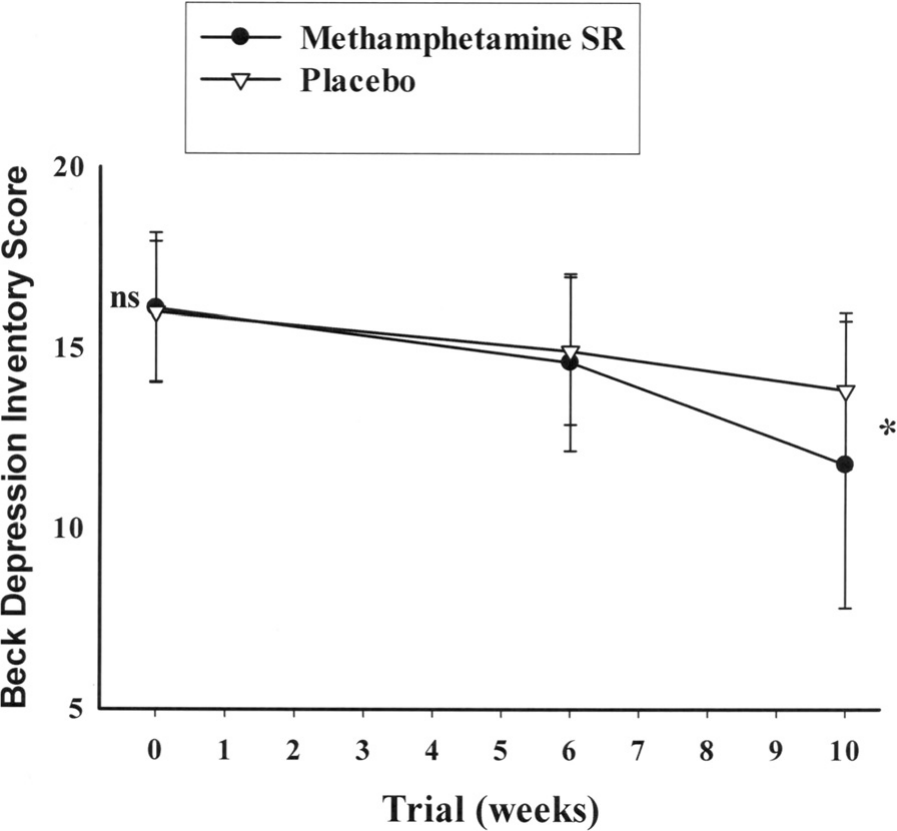
**Repeated measure for comparison of the effects of two treatments on Beck Depression Inventory Score.** Values represent mean ± standard deviation of the mean. P-values demonstrate the result of the independent T-test for comparison of scores between two treatment groups at each time point. NS indicates non-significant; and * = p < 0.05.

Assessment of the baseline *Depressive symptoms* scores did not reveal a significant difference between the treatment groups [MD (95% CI) = -0.10(-1.18-0.97), t(54) = -0.19, p = 0.84]. By the end of the trial, MPH-SR group showed significantly greater improvement in the depressive symptom scores compared to the placebo group [MD (95% CI) = 2.03(0.31-3.75), t(54) = 2.37, p = 0.02]. The effect of time × treatment interaction was also significant for the depressive symptom scores [F = 4.32, p = 0.02] (Figure 4).

### Adverse events

No major adverse events or mortality occurred during the period of this trial. As summarized in Table 2, a total of 10 adverse events were recorded with no significant difference in their frequency between the two groups (Table 2).

**Table 2:** **Frequency of adverse events in the study groups**

**Averse events**	**Methylphenidate slow selease, n**	**Placebo n**	**P value**
**Anxiety**	3	3	ns
**Decreased appetite**	3	2	ns
**Depression**	3	5	ns
**Muscle aches**	5	7	ns
**Nausea**	3	3	ns
**Headache**	10	5	ns
**Irritability**	5	8	ns
**Stomachache**	3	2	ns
**Insomnia**	8	9	ns
**Dizzy**	5	6	ns

ns: non-significant.

## Discussion

This preliminary study evaluated efficacy and safety of once-daily sustained-release methylphenidate using a double-blind, placebo-controlled design. Currently, there are no approved pharmacological treatments for methamphetamine dependence. This randomized placebo-controlled trial showed that slow-release methylphenidate can successfully be used in order to reduce drug craving. Furthermore, patients who received methylphenidate had less positive urine tests and it seemed that methylphenidate may decrease use of methamphetamine. The results of this study showed that depression decreased significantly more in the methylphenidate arm compared with the placebo arm.

Our results were in accordance with some previous studies [32,41,42]. Tiihonen and his colleagues have compared methylphenidate with aripiprazol for treatment of methamphetamine dependence, however, their study ended prematurely due to unexpected results of interim analysis and all of their patients were intravenous drug abuser; yet their results have been promising. Solhi et al. showed that both methylphenidate and risperidone were useful for decreasing drug craving in patients but the duration of this study was relatively short (3 weeks) and perhaps because of this the study failed to show the superiority of methylphenidate.

Methylphenidate blocks the methamphetamine-induced dopamine release and this effect may also antagonize the rewarding and reinforcing effects of methamphetamine and its use in dependent patients [31]. In addition, methylphenidate also increases dopamine levels and therefore may also act as substitution treatment for methamphetamine use [34,43]. Methamphetamine also releases norepinephrine and norepinephrine is thought to contribute to the acute effects of amphetamine-type drugs for the treatment of methamphetamine addiction [44]. Methylphenidate blocks the norepinephrine transporter and these drugs could also block methamphetamine-induced norepinephrine release [45].

Our results were inconsistent with the Miles study [34]. Miles *et al*. failed to confirm the usefulness of methylphenidate for amphetamine/methamphetamine dependence, but the retention rate in their study was low. In studies with long duration the Miles study was 22 weeks), the low retention rate is a major problem and one cannot necessarily conclude that all dropped out patients had experienced relapse. On the other hand, some studies show that amphetamine use began to decrease substantially as a function of time after 10 weeks of methylphenidate treatment reaching statistical significance at 18 weeks, which indicates that it may take an even longer period of time than 20 weeks to achieve full benefit from this treatment [32]. In these studies the participants were IV drug users which represented a more severe subtype of substance dependence. However, our participants used methamphetamine by smoking (the most common method of methamphetamine abuse in Iran) and perhaps because of this we could show the efficacy of methylphenidate in a shorter period of time. In the Konsteniusa study, sustained release methylphenidate could not affect craving for amphetamine or retention in treatment [46]. This study was carried out on abstinent persons, but in our study participants were active users. In terms of safety, the frequency of adverse events was not significantly different between the two study arms during this study. However, the present trial design was not particularly qualified for assessing the safety profile of MPH-SR, a point which merits further attention. This study has some limitations. The sample size was relatively small. Larger studies are required to replicate our findings. The effectiveness of methylphenidate beyond 10 weeks of treatment remains unknown.

## Conclusion

Sustained-release methylphenidate was safe and well-tolerated among active methamphetamine users and significantly reduced methamphetamine use, craving and depressive symptoms. This randomized placebo-controlled trial showed that sustained-release methylphenidate can successfully be used for treatment of methamphetamine dependence.
